# WHY AI GOVERNANCE SHOULD BE A FOCAL ISSUE FOR GERONTOLOGY

**DOI:** 10.1093/geroni/igae098.1111

**Published:** 2024-12-31

**Authors:** Clara Berridge, Clara Berridge

**Affiliations:** Chair; Discussant

## Abstract

Artificial intelligence (AI) and AI-mediated decision making currently impact older adults in numerous ways. From algorithmic decision making used in health insurance, employment, housing, and public benefits, to the information ecosystem, to social robots, AI applications implicate important issues of ethics, access, and ageism. The World Health Organization reports that the diverse interests of older adults are not well represented in discourses about AI policy making or governance. As AI harms are better understood, the call from public interest groups and from many academic fields has gotten louder for comprehensive AI and data privacy policy in the United States (U.S.), which lags behind other countries. Drawing from the international insights of European, Canadian, and U.S.-based research, this symposium will consider how older adults and their rights are represented in AI policy documents, laws, and policy discourse. Dr. Ho will discuss ethical concerns resulting from regulatory gaps in direct-to-consumer AI health monitoring platforms in the U.S context. Dr. Stypinska will highlight how European national (Germany, Spain, UK, Holland and Poland) and international policy documents related to AI position older adults. Dr. Gallistl will discuss the relevance of AI explainability in later life, as one of the key terms that currently inform EU AI-governance. Dr. Robillard will review findings and gaps from an analysis of international policies for social robots with aging applications. Participants with and without prior policy knowledge or AI research experience are welcome. Technology and Aging Interest Group Sponsored Symposium

